# Analgesic Effects of a Standardized Biofavonoid Composition from *Scutellaria baicalensis* and *Acacia catechu*

**DOI:** 10.3109/19390211.2012.708713

**Published:** 2012-08-10

**Authors:** Mesfin Yimam, Lidia Brownell, Mandee Hodges, Qi Jia

**Affiliations:** Unigen Pharmaceuticals Inc., Lacey, Washington, USA

**Keywords:** Acacia catechu, infammation, pain, Scutellaria baicalensis

## Abstract

Anti-infammatory properties of both baicalin and catechins have been widely reported. However, the reports of analgesic effects of baicalin and catechins are limited. Three commonly used pain-related animal models were employed to evaluate the analgesic activity of UP446, a standardized biofavonoid composition of baicalin and catechins. Carrageenan-induced paw edema, formalin test, and abdominal constriction assays were used to evaluate antinociceptive activity of 150 mg/kg or 100 mg/kg oral doses of UP446. Ibuprofen was used as a reference compound in each test. Pretreatment of carrageenan-induced hyperalgesic animals with UP446 at 150 mg/kg oral dosage reduced the hypersensitivity of pain by 39.5%. Similarly, a single dose of UP446, given orally at 100 mg/kg, exhibited 58% and 71.9% inhibition in pain sensitivity compared to vehicle-treated control in writhing and formalin tests, respectively. These fndings suggest that the standardized anti-infammatory biofavonoid composition, UP446, could also be employed to inhibit nociception.

## INTRODUCTION

Current use of prescription and over the counter non-steroidal anti-infammatory drugs (NSAIDs) has been limited due to untoward gastrointestinal and cardiovascular-related side effects. UP446, a standardized biofavonoid composition with primarily baicalin from the roots of *Scutellaria baicalensis* (*S. baicalensis*) and (+)-catechin from the heartwoods of *Acacia catechu* (*A. catechu*), has been used in both over the counter joint care dietary supplements and a prescription medical food. This natural favonoid composition reduces production of eicosanoids through inhibition of cyclooxygenase-1 (COX-1), cyclooxygenase-2 (COX-2), and 5-lipoxygenase (5-LOX) enzymes (Burnett, Jia, Zhao, & Levy, 2007) and also decreases expressions of inducible nitric oxide synthase (iNOS), nuclear factor-kappaB (NF-kB), and tumor necrosis factor-alpha (TNF-α) (Altavilla et al., 2009).

Augmented numbers of animal models have been developed in order to study nociception and the action of various analgesic drugs in animals. Most of these tests have overlapping mechanisms of action in pain perception in response to irritants. All models share hyperalgesia consistent with the action of prostaglandins for local peripheral sensitization and centrally mediated nociception pathways (Ferreira, Lorenzetti, & Correa, 1978). However, it is not uncommon to fnd bradykinin, histamine, and serotonin, as well as many other vasoactive substances that are released in acute infammatory responses. Intraplantar injection of carrageenan into rat hind paw produces infammation characterized by increased edema and plasma protein exudation with neutrophil extravasations and elevated metabolites of arachidonic acid from cyclooxygenase (COX) and lipoxygenase pathways (Gamache, Povlishock, & Ellis, 1986). Research has also shown that carrageenan-induced infammation in rats is associated with distinct phases in the infammatory process. The initial phase lasting for 30–60 min after carrageenan injection is dominated by the release of histamine, serotonin, and kinins and then mediated by prostaglandin and leukotrienes, which act relatively late in the development of infammation response. NSAIDs are known to prevent hyperalgesia of infammation by blocking the prostaglandin pathway (Flower, 1974).

Intraplantar injection of formalin is a commonly used model for acute infammatory pain, in which rodents display spontaneous nociceptive behaviors comprised of finching or licking or biting of the affected paw in two distinct phases (Dubuisson and Dennis, 1977). The two separate phases possibly show presence of two different types of pain. These characteristic periods of intense paw licking can be observed for the frst 5 min for the early phase with the late phase lasting from 20 to 40 min after the injection of formalin. It is suggested that the early phase pain is due to a direct effect of formalin on nociceptors and that proinfammatory prostaglandins do not play an important role during this phase. The late phase pain is believed to be as a result of infammation (Dubuisson & Dennis, 1977).

Acetic acid-induced visceral pain in mice has long been used for screening of narcotics and NSAIDs-like compounds for their antinociceptive effects. It is a nonspecifc model, which involves local peritoneal receptors. The abdominal constrictions elicited by mice consist of contractions of the abdominal muscle that progress posteriorly and usually end with simultaneous fexor extension of both hind limbs with arching of the back. Upon intraperitoneal administration of the phlogistic agent behavioral response lasts for 30 min (Collier, Dinneen, Johnson, & Schneider, 1968; Moore, Oluyomi, Babbedge, Wallace, & Hart, 1991; 2000).

Biofavonoids isolated from plants have been extensively reported as anti-infammatory compounds. Nevertheless, scarce data are available for their analgesic usage. For instance, aqueous extract of *Acacia karroo* stem bark in carrageenan and writhing animal model (Adedapo, Sofdiya, Masika, & Afolayan, 2008), butanolic fraction of dried leaves of *Acacia pennata* in writhing and formalin mouse model (Dongmo, Nguelefack, & Lacaille-Dubois, 2005), baicalin in carrageenan evoked thermal hyperplasia (Chou, Chang, Li, Wong, & Yang, 2003), myricitrin and gossypin in writhing mouse model (Meotti et al., 2006; Viswanathan, Sambantham, Reddy, & Kameswaran, 1984), Oligomeric procyjanidin, rutin, hyperoside, and quercetin in hot plate test (Rylski, Duriasz-Rowinska, & Rewerski, 1979) have shown variable degrees of analgesic activity. Dose-dependent antinociceptive activity was also observed in acacia honey-treated mice in tail-fick and paw-withdrawal tests (Azim, Perveen, Mesaik, & Simjee, 2007). Similarly, in structure-activity study, some methoxy derivatives of favone showed antinociceptive effect in acetic acid writhing and tail-fick mouse models (Thirugnanasambantham et al, 1993).

The anti-infammatory properties of both baicalin and catechins have been widely reported. However, the reports of analgesic effects of baicalin and catechins are limited. The primary objective of this study is to document the analgesic ability of a standardized biofavonoid composition with a combination of baicalin and catechins on three different animal models.

### Statistical Analysis

The results are represented as mean ± SD and percent changes. Statistical signifcance between two groups was calculated by means of analysis of variance followed by student's *t*-test. Results were considered to be statistically signifcant if *P* ≤ .05.

## MATERIALS AND METHODS

### Preparation of UP446

Detailed method for preparation of the two major favonoids, baicalin and catechin, from the roots of *S. baicalensis* and the heartwoods of *A. catechu*, respectively, were disclosed in a US patent (Jia, 2007). Briefy, *S. baicalensis* extract from roots was extracted with water and then recrystallized. The *S. baicalensis* extract contained baicalin as the major biofavonoid at content not less than 75% as well as other minor free-B-ring favonoids such as wogonin-7-O-G-glucuronide, oroxylin A-7-O-G-glucuronide, and baicalein. Catechin extract was obtained from repeated crystallization of an aqueous extraction of the heartwoods of an India medicinal plant, *A. catechu.* (+)-Catechin is the major component in the *A. catechu* extract with a content of not less than 65% plus a minor amount of its enantiomer, epicatechin, as well as other minor amounts of favans.

Analyses of the extracts were performed separately by two high-performance liquid chromatography (HPLC) methods. The quantifcation results of baicalin, from the *S. baicalensis* extract and catechin from the *A. catechu* extract were calculated by comparison HPLC peak area with known standards. The fnal UP446 composition was a mixture of *S. baicalensis* and *A. catechu* standardized extracts at a ratio 4:1 with baicalin content not less than 60% and catechin content not less than 10%. Other minor favonoids, such as wogonin 7-glucuronide and baicalein, account for about 15% of total weight. Moisture, ash, fat, and fiber constitute the remainder weight. The combined flavonoid content was analyzed by HPLC using a Phenomenex Luna 5μ C-18, 250 mm × 4.6 mm with a C-18 Security Guard cartridge in a column oven at 35°C. The mobile phase had a fow rate of 1.0 ml/min and used an isocratic 1% phosphoric acid:acetonitrile ratio of 85%:15% for the first 7 min and then a new gradient to 10%:90% from 7 min to 16.5 min and then an isocratic 1% phosphoric acid:acetonitrile gradient with a ration of 85%:15% for 7.5 min. The flavonoids were detected using a UV detector at 275 nm and identifed based on retention time by comparison with known flavonoid standards.

### Animals

Purpose bred male CD-1 mice (7–9 weeks), weighing 25–30 g and male Wistar rats weighing 145–160 g were purchased from US Department of Agriculture (USDA) approved laboratory animal vendor (Charles River Laboratories, Inc., Wilmington, MA). Animals were acclimated upon arrival for a week before being assigned randomly to their respective groups. Mice (5/cage) and rats (3/cage) were housed in a polypropylene cage and individually identifed by numbers on their tail. Each cage was covered with wire bar lid and fltered top (Allentown, NJ). Individual cage was identifed with a cage card indicating project number, test article, dose level, group, and an animal number. The Harlan T7087 soft cob beddings were used and changed at least twice weekly. Animals were provided with fresh water and rodent chow diet # T2018 (Harlan Teklad, 370W, Kent, WA) ad libitum and were housed in a temperature controlled room (22.2°C) on a 12 hr light-dark cycle. All animal experiments were conducted according to institutional guidelines congruent with guide for the care and use of laboratory animals.

### Carrageenan-Induced Inflammation

Local inflammation was induced by intraplantar injection of carrageenan λ (Sigma, St. Louis, MO; 100 µl of 1% [w/v] in saline) into the plantar surface of right hind paw of sedated rat (with 2.5% isofurane) at time 0 (*T* = 0) (Gamache et al., 1986; Guay, Bateman, Gordon, Mancini, & Riendeau, 2004; Huang, Lee, & Yang, 2006; dos Santos, Almeida, Lopes, & de Souza, 2006). Rats were placed under an inverted plexiglass cages on a wire mesh rack and allowed to acclimate for 20–30 min before each measurement was taken. Allodynia was evaluated by measuring responsiveness to a semifexible tip applied through the mesh foor perpendicular to the central plantar surface of the right hind paw. The tip was gradually applied with suffcient force to cause slight buckling of the flament against the paw. A positive response to the applied tactile pressure, noted by sharp withdrawal of the paw, was recorded automatically by an electronic Von Frey Anesthesiometer (2390 series Electrovonfrey, IITC, Woodland Hills, CA) (Vivancos, et al., 2003). Mechanical allodynia was evaluated before carrageenan inoculation, and thereafter 1 hr, 2 hr, 4 hr, and 6 hr. Paw edema was measured with the use of Plethysmometer (IITC, Woodland Hills, CA; Model 520) at time 0 (before carrageenan), 1 hr, 2 hr, 4 hr, and 6 hr after carrageenan injection. Animals (*N* = 12 per group) were orally gavaged with a positive control ibuprofen (Sigma, St. Louis, MO; lot # 037K1345) (150 mg/kg); test articles: UP446 (Unigen, Lacey WA; lot #UV07080) (150 mg/kg), and vehicle control (propylene glycol) 30 min before carrageenan induction.

### Nociceptive Behavior Elicited by Intraplantar Injection of Formalin in Mice

Mice (*N* = 12 per group) were habituated under an inverted Plexiglas observation chamber for 30 min to allow them to acclimatize to their surroundings. Animals were treated orally with 100 mg/kg of UP446, 100 mg/kg of ibuprofen, or vehicle control (propylene glycol) 30 min before intraplantar injection of formalin (20 µl of 2.5% solution) into the right hind paw of restrained mice using Hamilton syringe (Hamilton Company, Reno, Nevada, cat # 89520–0012) (Badilla, Mora, Lapa, & Emim, 1999; Dubuisson & Dennis, 1977; Kanegusuku et al., 2007; Tjolsen, Berge, Hunskaar, Rosland, & Hole, 1992; Zhang, Li, Zhang, Ding, & Du, 2007). Mice were immediately transferred to their individual observational chamber. The duration of time spent finching and/or licking of the infamed hind paw was monitored and recorded over a period of 60 min in a 10 min time block. Mirrors positioned behind the chambers enabled observation of the right hind paw when it was obscured from direct view.

### Visceral Pain Perception (Writhing Test)

Mice (*N* = 12 per group) were habituated under an inverted Plexiglas observation chamber for 30 min to allow them to acclimatize to their surroundings. Animals were treated orally with 100 mg/kg of UP446, 100 mg/kg of ibuprofen, or vehicle control (propylene glycol) 30 min before intraperitoneal administration of freshly made acetic acid solution (0.7% in 0.9% NaCl) at 10 ml/kg using 26 gauge needle syringes. The experiment was carried out at room temperature. After the challenge, each animal was placed back into its own individual section of the observation chamber and the number of constriction of the abdominal muscle together with stretching was counted cumulatively over a period of 30 min.

### Dose Selection

A dose of 150 mg/kg for carrageenan-induced infammation and 100 mg/kg for formalin and visceral pain perception tests were selected after a series of dose-response curve studies (data not shown) in respected models. UP446 performed better at the disclosed doses and was reproducible.

## RESULTS

### Carrageenan-Induced Hyperalgesia

The intraplantar injection of carrageenan in rats led to time-dependent increase in paw volume that was maximal after 4 hr and remained elevated thereafter. As shown in Table 1, animals treated with an oral dose of 150 mg/kg UP446 showed 37.8%, 39.5%, 30.7%, and 33.3% reduction in pain sensitivity at 1 hr, 2 hr, 4 hr, and 6 hr, respectively, when compared to the vehicle control animals. These percentage reductions were statistically signifcant at each time point analyzed against vehicle control (Table 1). The positive control ibuprofen showed statistically significant 20.3%, 39.8%, 32.6%, and 45.1% reduction at 1 hr, 2 hr, 4 hr, and 6 hr time points, respectively, compared to vehicle control (Table 1). However, when UP446 and ibuprofen data were compared, no statistically signifcant difference in inhibition was observed (*P* = .19 for 1 hr, *P* = .95 for 2 hr, *P* = .87 for 4 hr, and *P* = .22 for 6 hr).

**Table 1:** Antihyperalgesia Activity of UP446 on Carrageenan-Induced Paw Edema in Rats

	% Decrease of Vehicle				Mean ± SD (P-values)*			
		
Groups	1 hr	2 hr	4 hr	6 hr	1 hr	2 hr	4 hr	6 hr
Vehicle	–	–	–	–	23.8 ± 4.7	27.6 ± 5.4	30.3 ± 6.3	29.7 ± 6.3
Ibuprofen (150 mg/kg)	20.3	39.8	32.6	45.1	19.0 ± 4.9 (.02)	16.9 ± 6.7 (.0003)	20.4 ± 6.4 (.001)	16.3 ± 4.8 (.00001)
UP446 (150 mg/kg)	37.8	39.5	30.7	33.3	14.8 ± 9.5 (.01)	16.7 ± 8.2 (.001)	21.0 ± 9.7 (.01)	19.8 ± 8.3 (.003)

Rats (*N* = 12) were given intraplantar injection of carrageenan λ (100 μl of 1%[w/v] in saline) into the plantar surface of right hind paw of sedated rats at time 0 (*T* = 0). Rats were treated orally with UP446 (150 mg/kg) or ibuprofen (150 mg/kg) 30 min before carrageenan inoculation. Hypersensitivity threshold was determined by subtracting 1 hr, 2 hr, 4 hr, and 6 hr individual values from their respective T0 value. Data are expressed as mean ± SD and percent reduction of vehicle.

* *P*-values are shown in parentheses.

**Figure 1:**
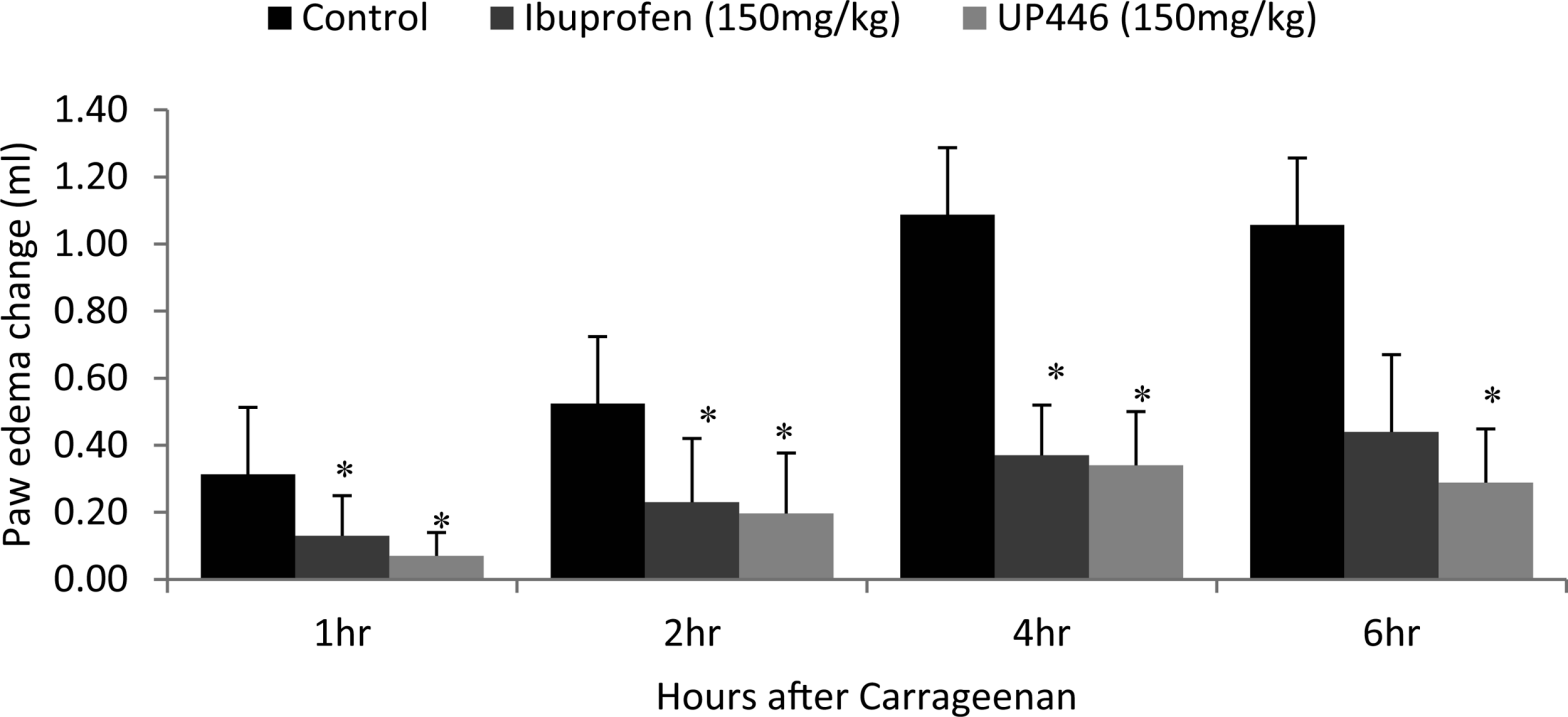
Effect of UP446 in carrageenan-induced paw edema: Male Wistar rats (*N* = 12) were given intraplantar injection of carrageenan λ (100 μl of 1% [w/v] in saline) into the plantar surface of right hind paw of sedated rats at time 0 (*T* = 0). Rats were treated orally with UP446 (150 mg/kg) or ibuprofen (150 mg/kg) 30 min before carrageenan inoculation. Paw edema was measured with the use of Plethysmometer at time 0 (before carrageenan), 1 hr, 2 hr, 4 hr, and 6 hr after carrageenan. Paw edema change was determined by subtracting 1 hr, 2 hr, 4 hr, and 6 hr individual values from their respective T0 value. Data are expressed as mean ± SD. ^*^P ≤ .05 vs. vehicle.

As seen in Figure 1, animals treated with UP446 showed up to 3.4, 1.6, 2.2, and 2.6-fold decreases in paw edema after 1 hr, 2 hr, 4 hr, and 6 hr of carrageenan injection compared to vehicle control. Similarly, animals that were administered ibuprofen showed 1.4, 1.3, 1.9, and 1.4-fold decrease in paw edema after 1 hr, 2 hr, 4 hr, and 6 hr of inocula compared to vehicle control (Figure 1). These fold reductions in paw edema for both ibuprofen and UP446 treatment groups were statistically significant at each time point considered except ibuprofen group at 6 hr.

### Formalin Test

Intraplantar injection of formalin (2.5%) in CD-1 mice elicited a biphasic nociceptive response compromising finching, leg raising, biting, and licking of the injected paw. Though previously reported formalin test-related antipain screenings limit their nociceptive reactions for the frst 40 min after formalin injections, we have plotted our observations in two time blocks, which included the duration between 20–40 min after formalin and 40–60 min after formalin. In this study, both time blocks showed greater than 50% reduction in pain perception for animals treated with single oral dose of 100 mg/kg UP446 or ibuprofen with statistical signifcance against vehicle control. During the late phase response, 20–40 min after injection of formalin, the duration of mentioned behavioral reactions was inhibited by 71.9% in animals pretreated with UP446 (Table 2). Ibuprofen attenuated the behavioral reaction to a similar extent of 72.9% (Table 2). No statistically signifcant difference in inhibition was observed when ibuprofen and UP446 data were compared (*P* = .91 for 20–40 min and *P* = .76 for 40–60 min).

**Table 2:** Effect of UP446 on Formalin Test in Mice

			(20–40) min		(40–60) min	
				
Groups	Dose (mg/kg)	*N*	Reaction Time (s)	Inhibition (%)	Reaction Time (s)	Inhibition (%)
Vehicle	0	12	129.7±7.9	–	72.5±5.2	
Ibuprofen	100	12	35.2±14.0	72.9**	26.4±9.6	63.6*
UP446	100	12	36.4±6.4	71.9**	30.9±22.6	57.4*

Biphasic (early and inflammatory phase) nociceptive response was elicited in CD-1 mice (25–30 g; *N* = 12) by intraplantar injection of formalin (2.5%) half an hour after test article. Mice were treated with UP446 or ibuprofen at 100 mg/kg orally. Data are expressed as mean ± SD.

** *P* ≤ .001;

* *P* ≤ .05 vs. vehicle.

### Visceral Pain Perception (Writhing Test)

**Figure 2:**
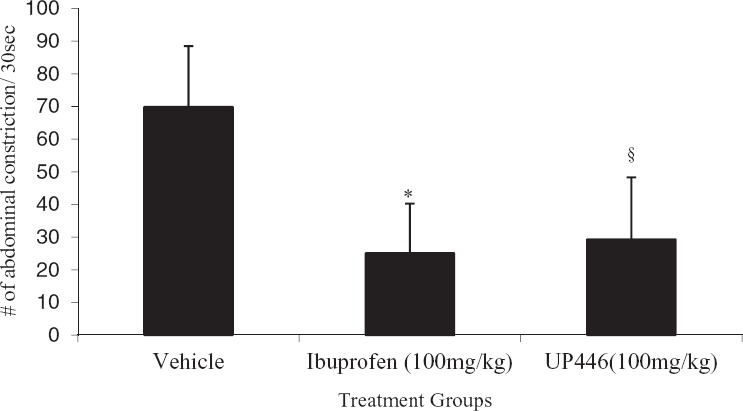
Effect of UP446in Writhing'stest: CD-1 mice were treated orally with 100 mg/kg UP446, ibuprofen, or vehicle control 30 min before intraperitoneal administration of freshly made acetic acid (0.7% in 0.9% NaCl) at 10 ml/kg. Mice treated with a single oral dose of 100 mg/kg of UP446 showed 58.0% reduction in abdominal muscle constriction compared to vehicle-treated animals. Comparable inhibition, 64.1%, was also observed for ibuprofentreated animals. Data are expressed as mean ± SD. *P ≤ .000001, ^§^*P* ≤ .00001 vs. vehicle.

Intraperitoneal injection of acetic acid solution (0.7%, 10 ml/kg) resulted in 69.9 ± 18.7 abdominal contractions in a period of 30 min. Mice treated with a single oral dose of 100 mg/kg of UP446 showed 58.0% reduction in abdominal muscle constriction compared to vehicle-treated animals (Figure 2). Comparable inhibition was also observed for ibuprofen-treated animals (i.e., 64.1% reduction) (Figure 2). No statistically signifcant difference in inhibition was observed when ibuprofen and UP446 data were compared (*P* = .56).

## DISCUSSIONS

Anti-infammatory and analgesic activities of NSAIDs have been extensively employed for decades. The mechanism of action is widely believed to involve inhibition of COX-mediated biosynthesis of prostacyclin (PGI_2_) and prostaglandin E_2_(PGE_2_) (Moncada, Ferreira, & Vane, 1973). These prostanoids exhibit both proin-fammatory and pronociceptive activity by potentiating the biological effects of other proinfammatory mediators including kinins, histamine, and serotonin during acute infammation. Nevertheless, the side effects of these commonly prescribed or over-the-counter drugs in the gastric mucosa and cardiovascular system limit their clinical utilization (Wallace, McKnight, Wilson, Del Soldato, & Cirino, 1997). In this regard, the search for anti-infammatory and/or antinociceptive natural alternatives with reduced side effect is urgent and necessary.

UP446, a unique and well-defned biofavonoid composition standardized with two major active compounds, baicalin and catechin originated from *S. baicalensis* and *A. catechu*, respectively, reduces production of eicosanoids through inhibition of COX-1, COX-2, and LOX enzymes (Burnett et al., 2007) and also decreases gene and protein expressions of proinfammatory iNOS, NF-kB, and TNF-α (Altavilla et al., 2009). The safety profles of individual biofavonoids and the standardized composition, UP446, have already been reported (Burnett et al., 2007; Chou et al., 2003; Isbrucker & Burdock, 2006). Burnett et al. concluded in their study that daily administration of UP446 by oral gavage at dose levels of 0, 250, 500, and 1,000 mg/kg/day to rats for 90 days produced no evidence of toxicity. As a result, a dose of 1,000 mg/kg/day was identifed as the no observed adverse effect level (NOAEL). He also reported drowsiness, stomach upset, nausea, and irritability in 9 out of 29 subjects treated with UP446 at 250 mg/day and 6 out of 39 placebo subjects after 60 days of double blind placebo-controlled human clinical study.

In the current study, biofavonoid composition, UP446, exhibited antinociceptive activity in all three animal models and also reduced carrageenan-induced paw edema. The hyperalgesia and edema caused by the carrageenan are cardinal signs of infammation, which commonly considered as most reliable signs when assessing a compound with a potential anti-infammatory and antinociceptive activity. Carrageenan-induced acute infammation involves the synthesis and release of vasoactive substances at the injured site. The role of PGE2 in the carrageenan-induced edema test has been well established in the literature (Flower, 1974). The early released bradykinin and the late PGE2 in this model are believed to be responsible for the development of edema and also for the sustained pain that accompanies the infammatory reaction as they are able to sensitize primary afferent neurons (Tjolsen et al., 1992). Therefore, the analgesic and antiedema activity of UP446 observed in this study could partially attribute to the ability of these biofavonoid compounds to block COX-mediated biosynthesis of PGE_2_ and reduce proinfammatory cytokines. In agreement with our fndings, Chou et al. reported that the anti-infammatory and analgesic mechanism of baicalin may be associated with the inhibition of critical infammatory mediators, including reduction gene expressions of COX-2 enzyme and proinfammatory cytokines (Chou, et al. 2003). Other studies conducted in aqueous extract of Acacia karroo stem bark, a similar class of *A. catechu*, have also shown the antinociceptive activity of biofavonoids in carrageenan-induced rat pain model (Adedapo et al., 2008).

Nociceptive behavior elicited by intraplantar injection of formalin in mice is a valued and reliable model in evaluating analgesic properties of plant extracts. UP446 inhibited the formalin nociception in late phase (infammatory phase) by more than 50%. Consistent with our results, butanolic fraction of dried leaves of *Acacia pennata* (Mimosaceae), a similar class of *A. catechu*, tested on formalin mouse model, showed a signifcant inhibition in pain sensitivity induced by the phlogistic agent (Dongmo et al., 2005).

The visceral pain inficted by the intraperitoneal administration of acetic acid in mice has been employed as a tool for screening analgesic compounds. Immediately after injection, animals showed abdominal constrictions consisting of contractions of the abdominal muscle, which progressed posteriorly and ended with simultaneous flexor extension of both hind limbs with arching of the back. These behavioral responses observed for the duration of 30 min were reduced to 16.7 ± 12.7 and 21.8 ± 16.8 for UP446 and ibuprofen, respectively, to that of the vehicle control, i.e., 57.0 ± 19.4. Similar to the carrageenan and formalin models, both compounds showed a greater inhibition of nociception comparable to ibuprofen. However, the analgesic activity demonstrated in this model was not suffcient to indicate the central or peripheral activity of the compounds. Substantiating our fndings, signifcant reduction in visceral pain perception was documented for Acacia karroo (Adedapo et al., 2008), *Acacia pennata* (Dongmo et al., 2005), gossypin (Viswanathan et al., 1984), myricitrin (Meotti et al., 2006), methoxy derivatives of favone (Thirugnanasambantham et al., 1993) using acetic acid writhing mouse model.

Since this is the first study in its kind to examine the antinociceptive effect of defned bioflavonoid composition from *S. baicalensis* and *A. catechu* in a range of behavioral tests, relative comparisons with the results of other published data are currently unavailable. Nevertheless, the antihyperalgesia and antiedema effects exhibited by UP446 pretreated animals in all the models were as expected provided that it is a dual inhibitor of COX and LOX enzymes.

In conclusion, the results documented on carrageenan-induced mechanical hyperalgesia, formalin-induced hind paw licking, and acetic acid-induced abdominal constriction show that UP446 was effective in attenuating pain perception caused by infammation suggesting that the compound could possibly be used for its analgesic beneft supplementing its anti-infammatory activity. At least in these models, UP446 has also shown comparable effcacy in pain inhibition to ibuprofen. However, due to the complexity of peripheral pain perception and accompanied responses exhibited in these animal models, a direct extrapolation of data to human usage needs to be proven in future human clinical trial.
